# Miller-Fisher Syndrome in the Setting of Influenza A Infection

**DOI:** 10.7759/cureus.36336

**Published:** 2023-03-18

**Authors:** Sharon Afflu, Gage Bollinger, Steven R Wolfe, Benjamin Smolar

**Affiliations:** 1 Neurology, Lake Erie College of Osteopathic Medicine, Pittsburgh, USA; 2 Family Medicine, Allegheny Health Network, Pittsburgh, USA; 3 Neurology, Allegheny Health Network, Pittsburgh, USA

**Keywords:** ivig, anti-gq1b antibody, influenza a infection, post-infectious, guillain-barre syndrome, miller fisher syndrome

## Abstract

Miller-Fisher syndrome (MFS) is a rare, and milder, variant of Guillain-Barre syndrome (GBS) that is characterized by ophthalmoplegia, areflexia, and ataxia, with the additional possibility of limb weakness. There is not a specific demographic or common situation in which MFS is usually seen. This paper details a suspected case of MFS in a 59-year-old male with a concurrent influenza infection. He had been experiencing progressive flu-like symptoms a few days prior to the onset of his neurological symptoms, presenting to the hospital with diplopia and paresthesias of his extremities. His physical exam on admission revealed areflexia and gait instability, as well as oculomotor nerve palsies that were causing his diplopia. After running tests to rule out other possible causes of his presentation, along with having a positive influenza A test, he was diagnosed with MFS and started on intravenous immunoglobulin (IVIG). His symptoms resolved by the end of the treatment course. Based on his presentation and resolution of symptoms, this would be one of the few reported cases of MFS following influenza A infection.

## Introduction

Miller-Fisher syndrome (MFS) is one of four rare variants of Guillain-Barre syndrome (GBS), with the others being acute motor axonal neuropathy, acute motor, and sensory axonal neuropathy, and Bickerstaff brainstem encephalitis [1]. Both GBS and MFS are associated with bacterial or viral illness preceding neurological symptoms, with the most common contender for GBS being gastrointestinal illness caused by Campylobacter jejuni [1], and the causative infective agent are often unknown in MFS [2]. The main difference between MFS and GBS is the severity and constellation of symptoms. GBS is characterized by ascending flaccid paralysis of the extremities, as well as sensory and autonomic dysfunction [3]. MFS tends to be milder and has a descending pattern of neurologic involvement, the most common symptoms being ophthalmoplegia, ataxia, and areflexia [1,4]. Antibodies against GQ1b ganglioside are found in 80%-95% of patients with MFS [1,4]; GQ1b ganglioside is part of oculomotor nerve (CN III) myelin [1]. Anti-GQ1b antibodies are thought to directly affect the neuromuscular junctions between cranial nerves (CN) and ocular muscles [1], therefore, damage to CN III, IV, and VI is autoimmune in nature [2] and explains the primary finding of ophthalmoplegia. The mainstay of treatment for MFS is a course of IVIG. It is a product composed of antibodies prepared from donated blood, meaning the antibodies collected are diversified [5]. IVIG is often given to aid in acute episodes of autoimmune conditions, theoretically modulating the activation and effector functions of B and T lymphocytes, thereby neutralizing pathogenic autoantibodies [5,6]; however, the exact mechanism is unknown due to its complexity. Nonetheless, this treatment confers an excellent prognosis.

## Case presentation

The patient in this case was evaluated on April 4, 2022. He is a 59-year-old male who presented to the hospital with a cough, diplopia, and worsening numbness and tingling in his hands and feet. Prior to admission, he had been experiencing five days of malaise, with nausea, diarrhea, and bilateral leg aches in addition to his cough. His diplopia developed along with his cough two days before admission, with gait unsteadiness soon following. His past medical history was significant for left-sided facial shingles and subsequent Bell’s palsy one year prior to presentation, with residual left-sided facial numbness and weakness.

On initial examination, he appeared ill and had a persistent nonproductive cough. He had a midline-restricted leftward gaze bilaterally and a restricted right inferolateral gaze of his right eye. He had binocular diplopia that resolved with monocular vision. Of note, he did not have decreased visual acuity of monocular vision or pain with eye movements. The rest of the CN that did not innervate extraocular muscles were intact besides left CN V and VII, consistent with his history of facial shingles and Bell’s palsy. He described a subjective numbness in his hands and feet bilaterally despite normal sensation on physical exam. Muscle strength was 5/5 globally, while reflexes were absent. He denied neck pain, neck stiffness, dizziness, vertigo, bowel incontinence, or bladder incontinence. His Romberg test was positive, his gait was unsteady. The remainder of his neurological exam was normal.

His complete blood count and comprehensive metabolic panel were within normal limits. His lumbar puncture was unrevealing with increased glucose (87 mg/dL), normal protein (29 mg/dL), and increased lymphocytes (96%). His computed tomography (CT) scan of the brain without contrast, CT angiogram of the head, and magnetic resonance imaging of the brain with and without contrast were all unremarkable. He was tested for Lyme disease, syphilis, multiple sclerosis, and West Nile viruses, which were all negative. A respiratory viral panel was sent and was positive for influenza A. With this constellation of symptoms and a positive influenza test, MFS was placed higher on the list of differential diagnoses-which included inflammatory conditions, paraneoplastic syndrome, and myasthenia gravis- and anti-GQ1b antibody levels were sent. He completed a course of IVIG 0.4 g/kg/day for five days, which is the standard dosing for treatment of GBS [7] and was discharged on his eighth day of admission with mildly improved reflexes at 1+/4; his diplopia, extraocular muscle palsy, gait disturbance, and extremity numbness/tingling had resolved. Several days after discharge, the anti-GQ1b IgG antibody levels came back with a negative titer of <1:100. This finding likely would have been addressed at the outpatient appointment with a neurologist, with possibility of nerve conduction studies (NCS) to investigate further, but the patient decided not to follow up after demonstrating notable improvement with his primary care physician a few days after discharge. However, based on his clinical picture and his symptom resolution after receiving IVIG, it is highly likely that this was a case of MFS due to influenza A infection.

## Discussion

CSF findings of albuminocytologic dissociation, which is a normal cell count in the presence of elevated protein levels, is a hallmark of GBS and MFS at their peaks. This patient was not found to have that, but the MFS diagnosis could not be ruled out due to CSF typically being normal early in the disease course [8]. The differential diagnoses and workup for this patient were broad, but the deciding factor that ultimately pointed to MFS was the classic triad of symptoms after a positive influenza test. Another method of diagnosing MFS is through NCS, which would show an absent or reduced H reflex, the equivalent of a monosynaptic stretch reflex [8,9]. NCS was not performed or offered because the patient did not follow up after discharge, but the result of this study would not have changed clinical management.

A case report from 2012 expressed that their case was possibly the first reported MFS associated with upper respiratory tract infection [2], the infection being influenza A. In recent years we have seen a few case reports linking COVID-19 [3], which is both an upper and lower respiratory tract infection, to MFS. This report we are presenting is likely one of the few cases of MFS following influenza A infection.

Pertaining to treatment, immunotherapy such as IVIG or plasma exchange is the first line. IVIG is generally preferred over plasma exchange due to convenience, availability, and minimal adverse effects [8]. Previous case reports of MFS had eventual curative results with a five-day course of IVIG [2, 4], with dosing based on patient weight. Our patient was able to achieve near-complete remission of his symptoms, only having slightly diminished reflexes remaining by discharge. Patients who do not receive IVIG eventually do recover, though taking significantly longer than if they had received the infusion: the median time for symptom disappearance being one month for ataxia and three months for ophthalmoplegia [4]. Whether or not patients receive IVIG or plasma exchange, MFS has an excellent prognosis for recovery. Depending on the severity of symptoms at presentation as well as other comorbid factors, patients may need to be discharged to acute or subacute rehabilitation [8], but despite this will often achieve complete or near-complete recovery.

## Conclusions

MFS is a rare variant of GBS that presents with ophthalmoplegia, ataxia, and areflexia, and has been associated with an upper respiratory infection. While it is one of the milder variants, its symptoms can be debilitating. Recognizing these symptoms, especially in a patient presenting with an upper respiratory infection like influenza, can be more important than relying on anti-GQ1b antibody levels; they are not always positive and can take a long time to result. Recognition of the clinical picture can aid in decreasing morbidity by prompt administration of immunotherapy.
